# Amyloid-β and APP Deficiencies Cause Severe Cerebrovascular Defects: Important Work for an Old Villain

**DOI:** 10.1371/journal.pone.0075052

**Published:** 2013-09-05

**Authors:** Salvadore Luna, D. Joshua Cameron, Douglas W. Ethell

**Affiliations:** 1 Molecular Neurobiology, Western University of Health Sciences, Pomona, California, United States of America; 2 Graduate College of Biomedical Sciences, Western University of Health Sciences, Pomona, California, United States of America; 3 College of Optometry, Western University of Health Sciences, Pomona, California, United States of America; 4 College of Osteopathic Medicine of the Pacific, Western University of Health Sciences, Pomona, California, United States of America; Pomona College, United States of America

## Abstract

Alzheimer’s disease (AD) is marked by neuritic plaques that contain insoluble deposits of amyloid-β (Aβ), yet the physiological function of this peptide has remained unclear for more than two decades. Using genetics and pharmacology we have established that Aβ plays an important role in regulating capillary bed density within the brain, a function that is distinct from other cleavage products of amyloid precursor protein (APP). APP-deficient zebrafish had fewer cerebrovascular branches and shorter vessels in the hindbrain than wild-type embryos; this phenotype was rescued by treatment with human Aβ peptide, but not a smaller APP fragment called p3. Similar vascular defects were seen in zebrafish treated with a β-secretase inhibitor (BSI) that blocked endogenous Aβ production. BSI-induced vascular defects were also improved by treatment with human Aβ, but not p3. Our results demonstrate a direct correlation between extracellular levels of Aβ and cerebrovascular density in the developing hindbrain. These findings may be relevant to AD etiology where high levels of Aβ in the brain parenchyma precede the development of neuritic plaques and dense aberrantly-branched blood vessel networks that appear between them. The ability of Aβ to modify blood vessels may coordinate capillary density with local metabolic activity, which could explain the evolutionary conservation of this peptide from lobe-finned fish to man.

## Introduction

Alzheimer’s disease (AD) is the most common cause of dementia in the elderly that currently afflicts more than 5.2 million people in the USA, and 13 million worldwide [1]. A definitive pathological feature of AD is neuritic plaques in affected brain areas, which contain insoluble deposits of amyloid-β (Aβ) peptide. During the early prodromal stages of AD, levels of soluble Aβ rise in the parenchyma of the medial temporal gyrus--years before the appearance of plaques or significant neurodegeneration. Neuritic plaques were first described by Alois Alzheimer [2] more than 100 years ago and Aβ was discovered more than 25 years ago [3], however, the physiological (i.e. non-pathological) function of Aβ remains enigmatic. An understanding of normal Aβ function(s) during development and homeostasis will provide important insights into its role in AD.

**Figure 1:**
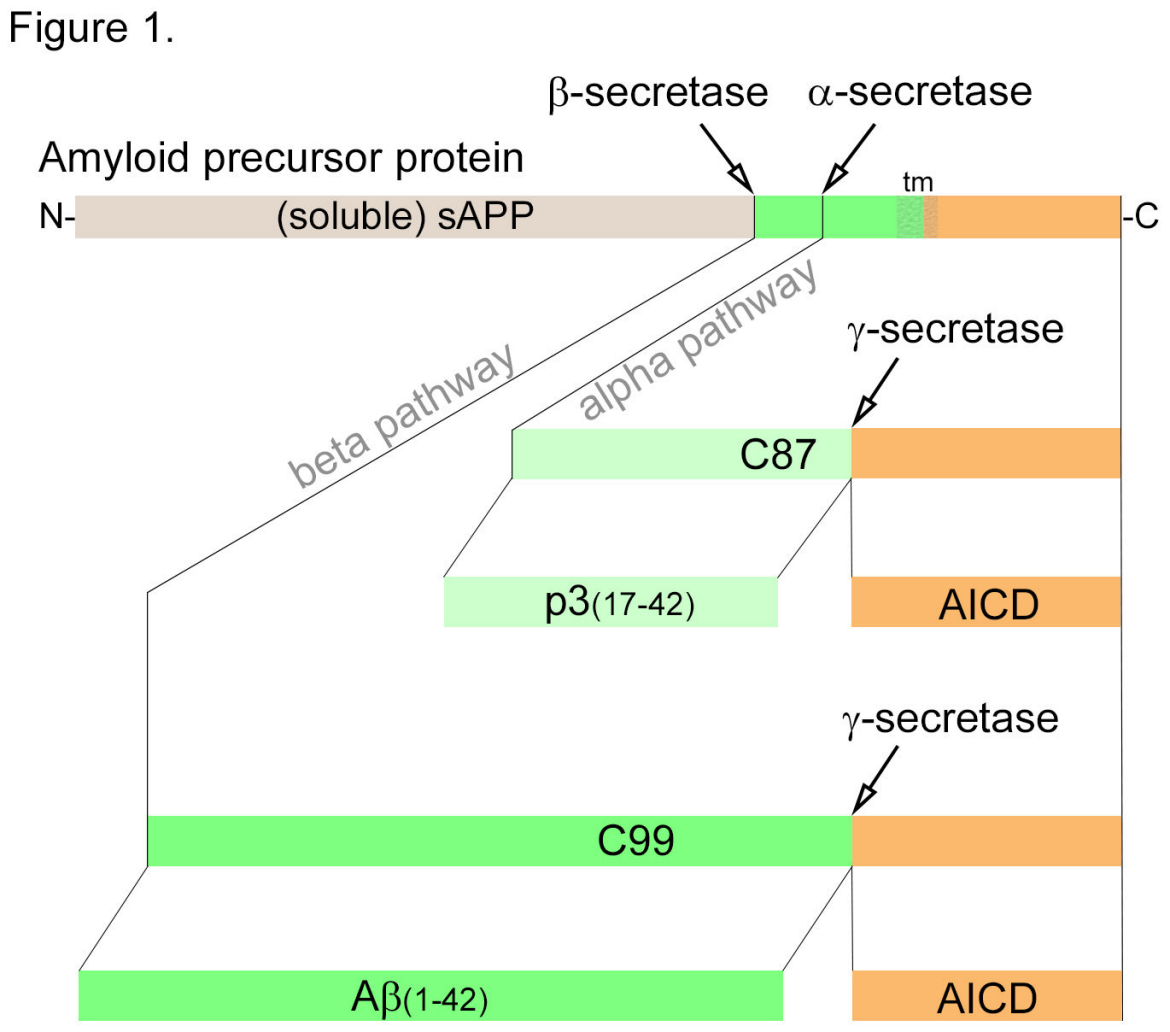
Schematic of APP processing that produces Aβ and p3 peptides. APP is initially cleaved by either α-secretase or β-secretases to yield a C87 in the alpha pathway, or C99 in the beta pathway, respectively. These cleavage events also produce an extracellular soluble APP (sAPP) fragment, from the amino terminus, that is slightly longer with α-secretase cleavage. The C87 and C99 fragments are subsequently cleaved within the transmembrane domains (tm) by γ-secretase to produce p3 and Aβ peptides, respectively. Both γ-secretase events produce an Aβ-intracellular-domain (AICD) fragment that is entirely cytosolic. Variability of γ-secretase cleavage on C99 produces Aβ fragments from 39–43 amino acids - only Aβ(1–42) is shown – and similar variability with C87 cleavage.

Aβ is produced throughout life by the brain, and other tissues, as a product of amyloid precursor protein (APP) proteolysis. Cleavage of APP by β-secretase produces a C99 fragment that is subsequently cleaved by γ-secretase to generate Aβ along with Aβ intercellular domain (AICD; Figure 1). Mutations in APP and subunits of the γ-secretase complex are the most common causes of early-onset AD [4].

The generation of rodent models for AD requires over-expression of human Aβ, as mouse and rat Aβ are less prone to aggregate due to three amino acid substitutions (Figure S1). While these sequence differences are notable as rodents are widely used experimental models, they are an exception to the rule. The amino acid sequence of human Aβ (1–42) is identical to that of most terrestrial vertebrates and lobe-finned coelacanth (Figure S1). Conversely, the amino acid substitutions in mice and rats occur only in the clade *Rodentia*. The high degree of evolutionary conservation, from the Devonian period to modern humans, suggests that Aβ plays an indispensable role in survival and reproductive fitness. This function must coincide with physiological aspects of vertebrate development and/or homeostasis as Aβ conservation does trace back to invertebrates.

We recently reported that high levels of Aβ increase cerebrovascular branching during embryonic development of the zebrafish hindbrain [5]. To determine whether lower levels of Aβ would have a reciprocal effect we employed genetic and pharmacological methods to generate APP- and Aβ-deficient zebrafish embryos, and then looked for cerebrovascular defects. We also tested if those defects could be rescued using human Aβ peptide or a shorter cleavage product of APP that corresponds to residues 17-42 of Aβ, called p3 (Figure 1).

## Results

**Figure 2:**
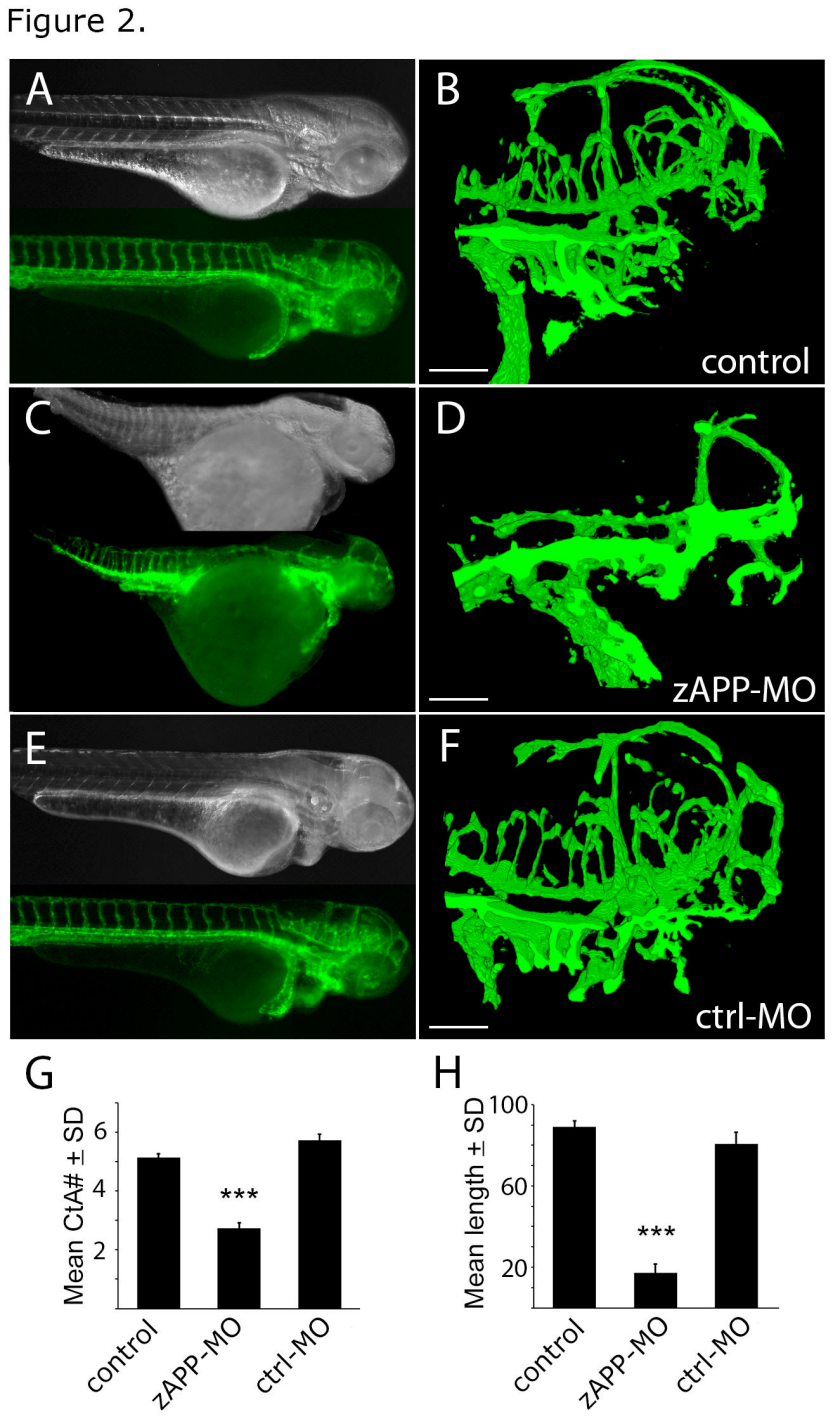
Cerebrovascular defects in APP-deficient zebrafish embryos. (A) Dark field (top) and fluorescence (bottom) images of a control transgenic embryo at 3 dpf shows vascular structures dues to EGFP expression in endothelial cells. (B) Confocal image (projected stack) of cerebrovascular structures in the head of the fish in *A*. (C) Dark field (top) and fluorescence (bottom) images of a zAPP-MO embryo at 3 dpf. (D) Confocal image (projected stack) of cerebrovascular structures in the head of the fish in *C*. (E) Dark field (top) and fluorescence (bottom) images of a ctrl-MO embryo at 3 dpf. (F) Confocal image (projected stack) of cerebrovascular structures in the head of the fish in *E*. (G) Graph showing the number of CtA branches in control (N = 30), zAPP-MO (N = 15), and ctrl-MO (N = 15) zebrafish at 3 dpf (***, *P* < 8.9e-16). (H) Mean CtA branch lengths in control (N = 28), zAPP-MO (N = 14), and ctrl-MO (N = 8) embryos at 3 dpf (***, P < 9.8e-23); scale bars = 100 μm.

APP-deficient zebrafish (zAPP-MO) were generated by injecting single cell embryos with APP-targeted oligonucleotides (morpholinos) that block the translation of APP mRNA for at least 3 days post-fertilization (dfp) [6]. zAPP-MO embryos had short bodies with large yolk sacs (Figure 2A, B). In vivo confocal imaging of cerebrovascular structures was made possible by a transgenic zebrafish line that expresses EGFP in vascular endothelial cells [7] (Figure 2A–F, Figure S2). APP-deficient embryos had significantly fewer central artery (CtA) branches off the primordial hindbrain channel (Figure 2C, D) than control embryos (Figure 2A, B) or embryos injected with a scrambled-sequence morpholino (ctrl-MO; Figure 2E–G; Table 1). Further, the mean length of CtA branches in zAPP-MO embryos was significantly shorter than in either control (Figure 2H; Table 2); indeed, the vessels in APP-deficient embryos were ~15% the length of wild-type.

**Table 1:** Mean CtA branch numbers of 3 dpf zebrafish embryos in all conditions examined.

**condition**	**N**	**mean**	**SEM**	**p value**
zAPP-MO	15	2.73	0.20	8.93E-16
zAPP-MO+Aβ	15	5.27	0.20	1
zAPPMO+P3	15	2.93	0.20	8.78E-14
BSI	29	4.24	0.14	0.000602
BSI+Aβ	8	4.88	0.27	1
BSI+P3	10	3.40	0.25	3.02E-07
Control	30	5.13	0.14	NA
Ctrl-MO	15	5.73	0.20	0.445238

*P* values were calculated by ANOVA with Bonferroni correction.

**Table 2:** Mean CtA branch length of 3 dpf zebrafish embryos in all conditions examined.

**condition**	**N**	**mean**	**SEM**	**p value**
zAPP-MO	14	17.20	4.42	1E-22
zAPP-MO+Aβ	10	87.04	5.23	1
zAPPMO+P3	14	34.62	4.42	1E-15
BSI	21	46.07	3.61	3E-13
BSI+Aβ	8	74.02	5.85	0.7282
BSI+P3	10	34.42	5.23	4E-13
Control	28	88.99	3.12	NA
Ctrl-MO	8	80.68	5.85	1

*P* values were calculated by ANOVA with Bonferroni correction.

**Figure 3:**
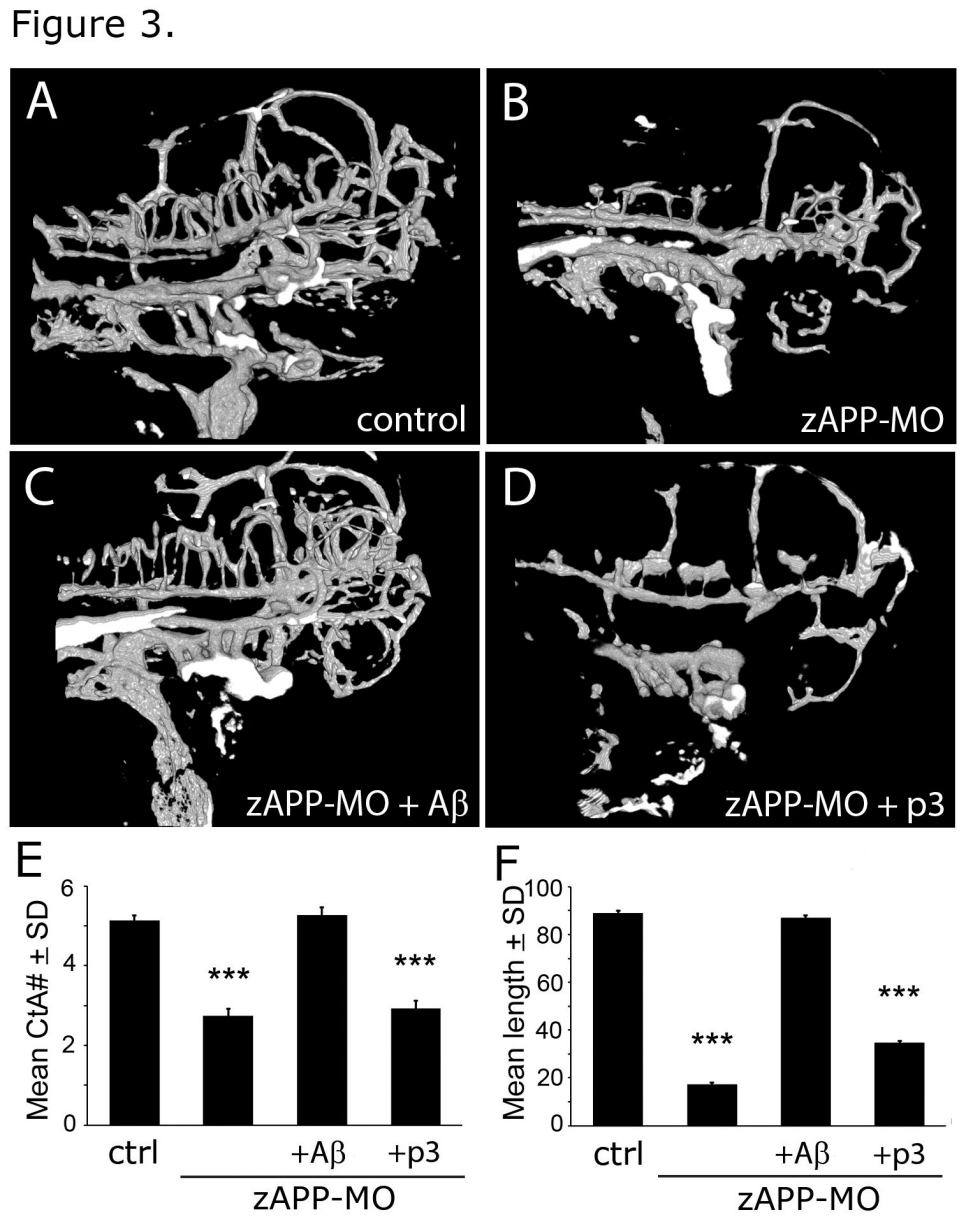
Aβ rescued vascular defects in APP-deficient (zAPP-MO) zebrafish embryos at 3 dpf. (A) Confocal image (projected stack) of a control zebrafish embryo at 3 dpf. (B) Comparable image of a zAPP-MO embryo at 3 dpf. (C) Cerebrovascular structures of an Aβ-treated zAPP-MO were similar to non-injected controls. (D) p3 treatment did not rescue vascular defects in zAPP-MO embryos. (E) Graph of CtA branch numbers in embryos in the control (N = 30), zAPP-MO (N = 15), Aβ-treated zAPP-MO (N = 15), and p3-treated zAPP-MO (N = 15) embryos at 3 dpf. Differences between control and zAPP-MO were significant (*P* < 8.9e-16), but there were no significant differences between Aβ-treated zAPP-MO and control or ctrl-MO. p3-treated zAPP-MO had significantly fewer branches than control embryos (*P* < 8.7e-14). (F) Graph of mean CtA branch lengths in control (N = 28), zAPP-MO (N = 14), Aβ-treated zAPP-MO (N = 10), and p3-treated zAPP-MO (N = 10) embryos at 3 dpf. Differences between control and zAPP-MO were significant (*P* < 9.8e-23), but there were no significant differences between Aβ-treated zAPP-MO and control or ctrl-MO. p3-treated zAPP-MO had significantly shorter vessel lengths than control embryos (*P* < 1.3e-15).

To determine whether vascular abnormalities in zAPP-MO embryos were primarily due to Aβ deficiency we treated them with human Aβ (1–42) starting at 2 dpf, when vascular abnormalities could be discerned in zAPP-MO embryos (Figure S3). Aβ treatment of zAPP-MO embryos significantly increased CtA branching, compared to untreated zAPP-MO embryos at 3 dpf (Figure 3A–C; Table 1). Moreover, mean vessel length was significantly longer in Aβ-treated zAPP-MO, in comparison to untreated zAPP-MO embryos (Figure 3F; Table 2). Rescue of the zAPP-MO vascular phenotype by Aβ peptide was so complete that CtA branch numbers and vessel lengths were not significantly different from uninjected control or ctrl-MO embryos (Figure 3E, F; Tables 1, 2). These findings establish that APP-deficiency reduces vascular branching and vessel length due to low levels of Aβ.

Aβ is a product of serial β- and γ-secretase cleavage of APP that generates 39-43 residue peptides in the “beta pathway”. By contrast, p3 is the result of serial α- and γ-secretase cleavage of APP to produce 23-27 residue peptides in the “alpha pathway” (Figure 1). The p3 peptide corresponds to residues 17-42 at the carboxyl end of human Aβ. While the vascular phenotype of zAPP-MO embryos was rescued by exogenous Aβ treatment, p3 peptide treatment had no significant effects on CtA branching or vessel length in zAPP-MO embryos (Figure 3D–F). This difference in the abilities of Aβ but not p3 to rescue vascular defects in APP-deficient embryos establishes that residues 1-16 are critical for the vascular activity of Aβ.

**Figure 4:**
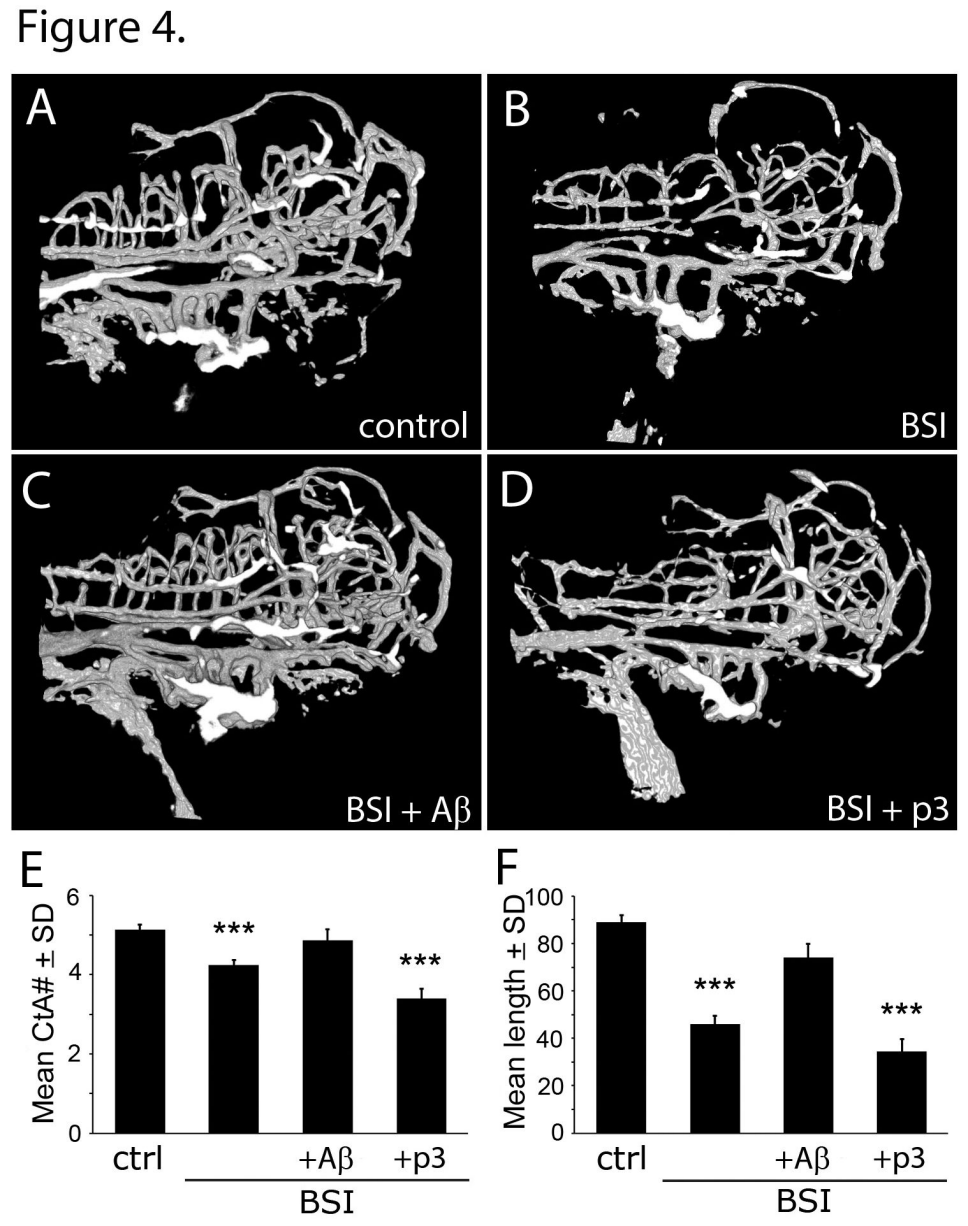
Aβ-deficiency induced by BSI-treatment caused vascular defects that were rescued by Aβ, but not p3. (A) Confocal image of cerebrovascular structures in an untreated control zebrafish at 3 dpf (control). (B) Vascular structures in a BSI-treated embryo at 3 dpf (BSI). (C) Cerebrovascular structures in a BSI-treated embryo that was treated with Aβ (BSI+Aβ) showed rescue of the vascular defects. (D) Vascular defects in a BSI-treated embryo were not rescued by p3 (BSI+p3). (E) Graph of CtA branch numbers in control (N = 30), BSI (N = 29), BSI+Aβ (N = 8), and Aβ+p3(N = 10) embryos at 3 dpf. Differences between control and BSI were significant (*P* < 0.0006), but there was no significant difference between BSI+Aβ and control embryos. BSI+p3 embryos had significantly fewer branches than control embryos (*P* < 3.0e-7). (F) Graph of mean CtA branch lengths in control (N = 28), BSI (N = 21), BSI+Aβ (N = 8), and BSI+p3(N = 10) embryos at 3 dpf. Differences between control and BSI were significant (*P* < 3.0e-13), but there was no significant difference between BSI+Aβ and control embryos. BSI+p3 embryos had significantly shorter vessel lengths than control embryos (*P* < 3.6e-13).

APP-deficient zebrafish embryos (zAPP-MO) lack full-length APP and all of its cleavage products, including Aβ, p3, soluble-APP and AICD (Figure 1). To provide conclusive evidence that the vascular defects in zAPP-MO embryos were due to low levels of Aβ we generated Aβ-deficient embryos using β-secretase inhibitor (BSI) to block endogenous production of the peptide. BSI-treated embryos had significantly fewer CtA branches than untreated control embryos (Figure 4A, B, E; Table 1). Vessel length in BSI-treated embryos was also shorter than in untreated controls (Figure 4F; Table 2). Interestingly, gross morphological aberrations in BSI-treated embryos were not as severe as with zAPP-MO embryos, in that they did not have a shortened body and their large yolk sacs were only slightly misshapen. This observation is consistent with non-vascular defects being caused by the loss of full-length APP or other cleavage product in zAPP-MO embryos.

To rescue the vascular defects in BSI-treated embryos we administered human Aβ starting at 2 dpf. Aβ treatment significantly increased CtA branch number, compared to embryos treated with BSI alone (Figure 4). Moreover, vessel length in BSI-treated embryos was also significantly longer in embryos that were also treated with Aβ (Figure 4F; Table 2). Importantly, p3 peptide did not rescue vascular defects in BSI-treated embryos (Figure 4B–F; Tables 1 & 2).

## Discussion

Through genetic and pharmacological manipulations we have established that Aβ deficiency reduces cerebrovascular branching and vessel length in embryonic zebrafish hindbrain. These findings support a physiological role for Aβ in capillary bed density and remodeling that has been conserved for most of vertebrate evolution. Taken together with findings from a previous report [5], these results demonstrate that β-secretase and γ-secretase inhibitors have complementary effects on cerebrovascular branching. Inhibiting either secretase will prevent Aβ production but there are side-effects with GSI’s due to other substrates that rely on γ-secretase for processing, including Notch, which explains why BSI and GSI have reciprocal effects on blood vessel branching even though they both inhibit Aβ production. Gamma-secretase inhibitors and elevated levels of soluble Aβ increase blood vessel branching in the embryonic zebrafish hindbrain [5]; however, APP-deficiency and BSI (Aβ-deficiency) reduced blood vessel branching and decreased vessel length. Remarkably, vascular defects in APP- and Aβ-deficient embryos were rescued by human Aβ. Although Aβ in zebrafish, and other *Teleosts*, varies from Aβ in most vertebrates, including man, its function remains consistent (Figure S1). Human Aβ is able to reverse vascular defects caused by the loss of endogenous APP and Aβ in zebrafish. This observation suggests that the coelacanth-to-human sequence of Aβ is at least as potent for this vascular function as zebrafish Aβ, and may represent the optimal form of Aβ. As a regulator of blood vessel branching and length Aβ plays a key role in regulating local capillary density. For example, we recently showed that human Aβ can increases capillary bed density in adult zebrafish retina [8]. Although Aβ availability affects vascular length and branching, neither higher nor lower levels significantly impacted overall embryo length (Figure S4).

The idea that Aβ availability impacts capillary density provides a new perspective of Aβ biology that is directly relevant to AD etiology. For example, the side-effects in recent clinical trials that targeted Aβ production to treat AD could be the result of interfering with this function [9–12]. Gamma-secretase inhibitor (GSI) trials were halted due to adverse effects [13] that included vasogenic edema [14], liver damage, and worst of all an exacerbation of AD progression [15]. Those adverse effects were attributed to the disruption of processes that rely on other γ-secretase substrates, such as Notch [16,17]. Notch is a major regulator of vascular stability and inhibitors of Notch signaling induce hyper-vascularization that remarkably similar to the dense aberrant blood vessel networks that form between Aβ-containing plaques [18]. Therefore, trials that attempt to block Aβ production with GSI’s trade the modest Notch blocking effects of Aβ for the more efficient Notch blocking effects of GSI, which accelerates neovascular changes that include in all brain regions [5,19].

If a major physiological function of Aβ is to increase capillary bed density and high levels of Aβ cause hyper-vascularization in AD, then BSI’s should reduce endogenous levels of Aβ and mitigate its effects on aberrant blood vessel formation--without the disastrous side-effects of GSI’s. These findings are particularly relevant as BSI clinical trials are currently underway. BSI’s hold promise as a therapeutic approach to AD, though it may be prudent to watch for high-dose effects on vascular pruning in brain areas not affected by AD pathology, and highly vascularized tissues such as lung and kidney.

## Materials and Methods

Tg (kdr:EGPF) s843 transgenic zebrafish, expressing GFP in vascular endothelial cells, were obtained from the Zebrafish International Resource Center/ZIRC (Eugene, OR), and maintained under standard conditions at 28.5°C on a 10 h dark -14 h light cycle [7,20]. Embryos were staged in hours post-fertilization (hpf) and days post-fertilization (dpf) based on morphological features. Embryos were raised in E3 buffer (5 mM NaCl, 0.17 mM KCl, 0.33 mM CaCl_2_, 0.33 mM MgSO_4_) at 28.5°C. Human Aβ1-42 was purchased from Biomer and prepared as previously described [21]. Human p3 corresponding to peptides 17-42 of Aβ was purchased from Anaspec, and prepared in the same way as Aβ.

### Ethics Statement

All animal husbandry and experiments were approved and conducted in strict accordance with recommendations in the Guide for the Care and Use of Laboratory Animals of the National Institutes of Health. The protocol was approved by the Western University of Health Sciences Institutional Animal Care and Use Committee.

### Embryo Treatments

Zebrafish were dechorionated at 24 hpf prior to treatment. Treatment solutions were diluted in E3 buffer containing 0.003% 1-phenyl-2-thio-Urea (PTU) to inhibit pigment development. Treatments consisted of 25 μg/mL monomeric Aβ1-42, 25 μg/mL p3, 20 μg/mL BSI, or E3 buffer for a negative control. Embryos remained in treatment conditions until 3 dpf when they were fixed in 4% paraformaldehyde/PBS overnight.

### Morpholino injections of embryos

Fertilized zebrafish embryos were collected and microinjected with ~4 nl of zAPP-MO morpholino at the single cell stage (<45 minutes post-fertilization), using a dissecting microscope and Drummond Nanoject II. Phenol red was included in the solution to monitor the injections.

### Zebrafish imaging

Eyes were removed from the fixed embryos by grazing the membrane with a tungsten needle. Zebrafish embryos were laid on their sides and mounted on coverglass using a solution of methylcellulose. Imaging was done with a Nikon C1 3-color confocal microscope using 10X or 20X objectives. Confocal slices were captured from the lateral surface of the head to midline. Image stacks were then converted to 8-bit, Gaussian smoothed, binarized to establish a threshold, and skeletonized to obtain centerlines for each path/branch. 3D renderings were created to obtain rotational volume images, movies, and for vascular data analysis [22]. Hindbrain vascular branch length was measured with the segmentation simple neurite tracer plugin in Fiji distribution of ImageJ to determine the dorsal distance traveled of the central arterial branches (CtA) from the posterior hindbrain channel (PHBC) bounded by the anterior medial cerebral vein (MCeV) and the posterior cerebral vein (PCeV). Branching of central arteries (CtA) emerging from the PHBC, which project to basilar artery, was done as previously described [5].

### Statistical analysis

Statistical comparisons between treatment groups were evaluated by one-way ANOVA with Bonferroni’s post-hoc test (Table 1). Data presented in all histograms represent mean and standard error of the mean, as detailed in Tables 1 and 2.

## Supporting Information

Figure S1 **Evolutionary alignment of Aβ1-42 from humans to cartilaginous fishes.** Text without background color indicates prefect conservation of the residue with human and coelacanth Aβ. Evolutionary times are not scaled.
(TIF)

Figure S2 **Movie of cerebrovascular structures in 3 dpf zebrafish.** 3D rendering of cerebrovascular structures in a 3 dpf zebrafish embryo with labels, generated from confocal imaging of EGFP fluorescence in vascular endothelial cells. Rotation of the rendering shows 3D relationships of cerebrovascular structures in the head on the left side of the fish. Transition to pseudocolor shows vessel diameter differences; compare gills at the bottom to CtA in the hindbrain. The next transition shows a skeletonized rendering of blood vessels. CtA vessels are highlighted with purple.
(MOV)

Figure S3 **Vascular abnormalities in zAPP-MO could be discerned in embryos at 2 dpf.** Fluorescence (top) and confocal (bottom) microscopy of 2 dpf embryos. (A) control uninjected embryo. (B) Embryo injected with scrambled sequence morpholino oligonucleotides (ctrl-MO). (C) Embryo injected with zAPP-targeting morpholino. Note CtA emerging from the lateral PHBC on the left side of A and B, but not C.
(TIF)

Figure S4 **Effects of Aβ availability on embryo size at 4 dpf.** Histogram showing total length of embryos treated under control condition, and in E3 water containing Aβ (5, 15, or 25 μg/ml). BSI (20 μg/mL), or GSI (10 μg/mL).
(TIF)
