# Supplementary figures and images for: Amyloid-β and APP Deficiencies Cause Severe Cerebrovascular Defects: Important Work for an Old Villain
Source: PLoS One. 2013 Sep 5;8(9):e75052. doi: 10.1371/journal.pone.0075052 (PMC3764155; doi:10.1371/journal.pone.0075052)

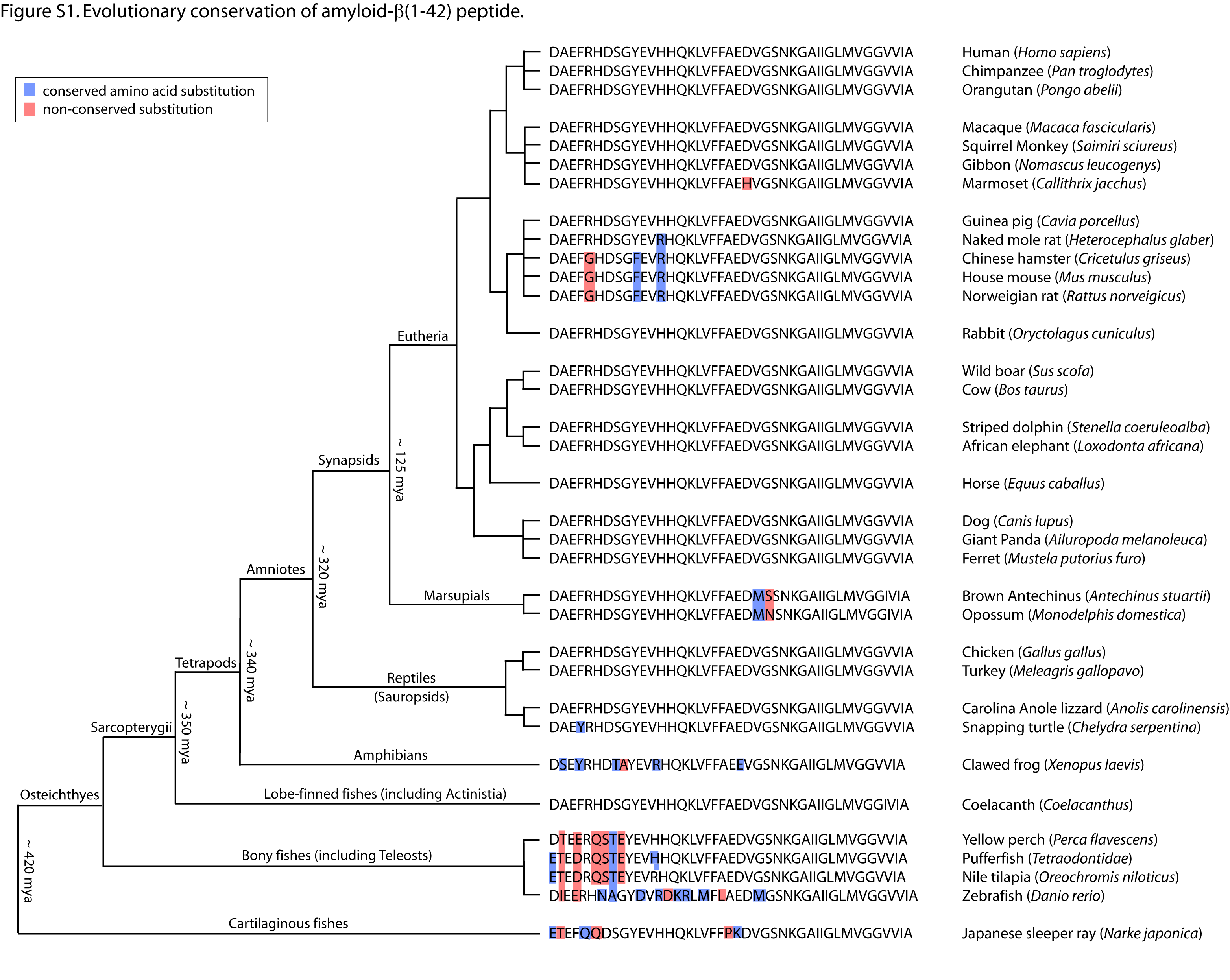

Supplement: Figure S1 — Evolutionary alignment of Aβ1-42 from humans to cartilaginous fishes. Text without background color indicates prefect conservation of the residue with human and coelacanth Aβ. Evolutionary times are not scaled. (TIF) [file pone.0075052.s001.tif]

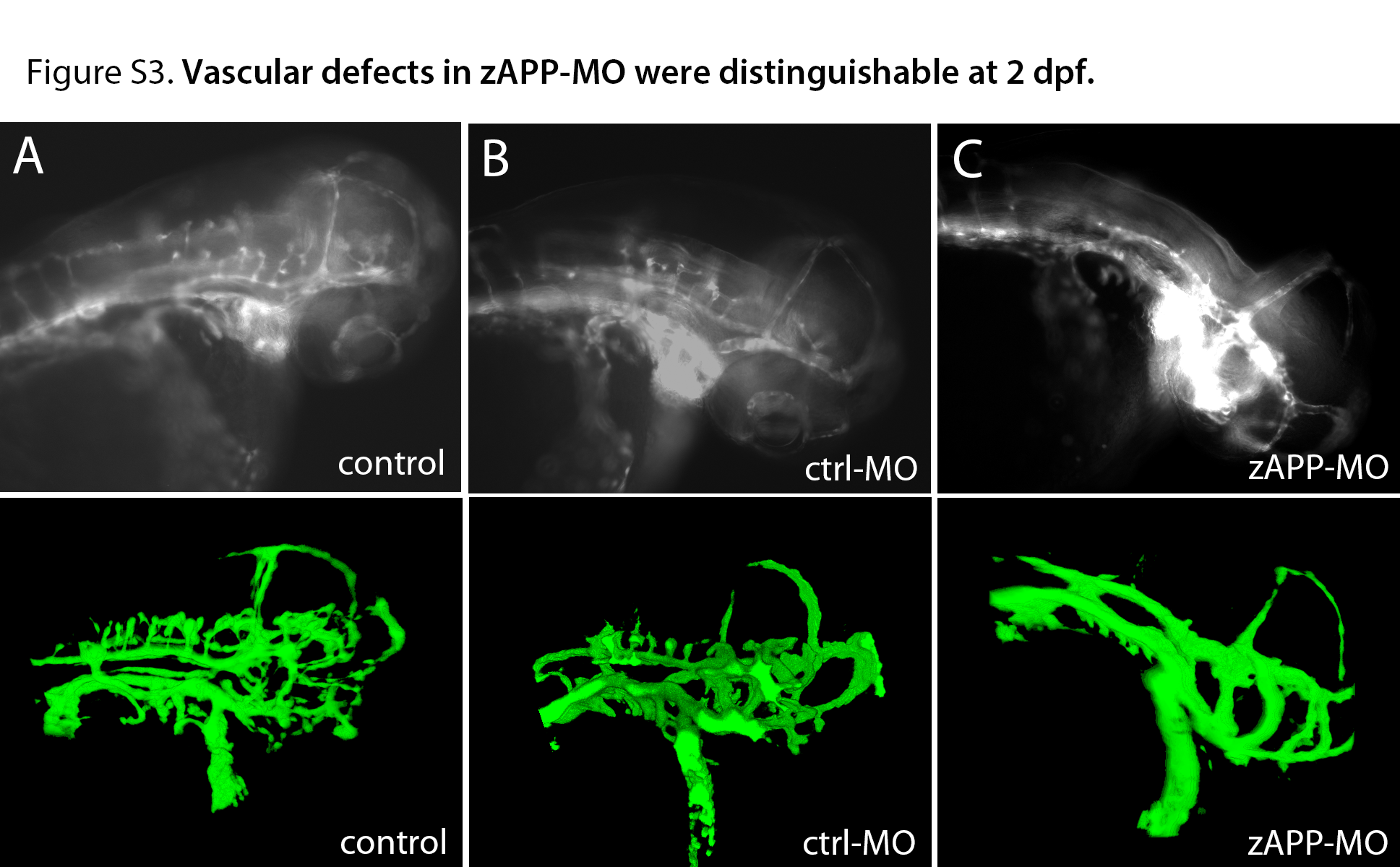

Supplement: Figure S3 — Vascular abnormalities in zAPP-MO could be discerned in embryos at 2 dpf. Fluorescence (top) and confocal (bottom) microscopy of 2 dpf embryos. (A) control uninjected embryo. (B) Embryo injected with scrambled sequence morpholino oligonucleotides (ctrl-MO). (C) Embryo injected with zAPP-targeting morpholino. Note CtA emerging from the lateral PHBC on the left side of A and B, but not C. (TIF) [file pone.0075052.s003.tif]

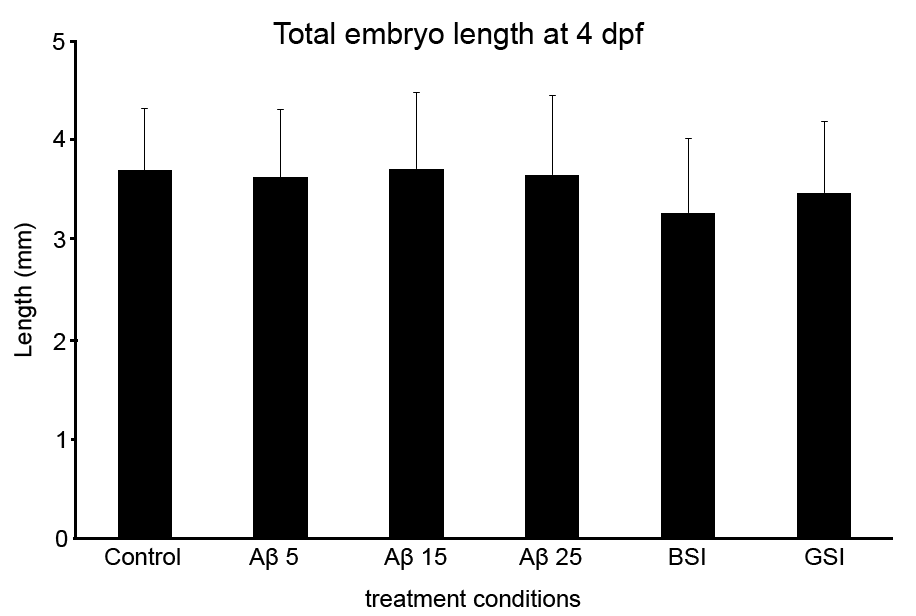

Supplement: Figure S4 — Effects of Aβ availability on embryo size at 4 dpf. Histogram showing total length of embryos treated under control condition, and in E3 water containing Aβ (5, 15, or 25 μg/ml). BSI (20 μg/mL), or GSI (10 μg/mL). (TIF) [file pone.0075052.s004.tif]
